# Decreased risk of secondary brain herniation with intracranial pressure monitoring in patients with haemorrhagic stroke

**DOI:** 10.1186/1471-2253-14-19

**Published:** 2014-03-21

**Authors:** Jingsong Zeng, Ping Zheng, Wusong Tong, Weimin Fang

**Affiliations:** 1 Department of Neurosurgery, Shanghai Pudong New area People’s Hospital, 490 South Chuanhuan Road, Shanghai, China

**Keywords:** Intracranial-pressure monitoring, Hematoma enlargement, Brain herniation, Hemorrhagic stroke

## Abstract

**Background:**

Intracranial-pressure (ICP) monitoring is considered standard care for severe traumatic brain injury and is used frequently, but the efficacy of treatment based on monitoring in patients with hemorrhagic stroke has not been rigorously assessed. In this study, we investigated the clinical value of ICP monitoring in patients with hemorrhagic stroke.

**Methods:**

We conducted a randomized, unblinded, controlled trial in which 90 patients with hemorrhagic stroke were randomly assigned to ICP monitoring or a control group. The primary outcome was a composite of incidence rate of hematoma enlargement and secondary brain herniation. The secondary outcome was neurological status assessed using the Glasgow Outcome Scale scores at 6 months post-onset. Characteristics of the patients at baseline and outcome measurements were also compared between the two groups.

**Results:**

There was no significant between-group difference in the incidence of hematoma enlargement (control group, 38.6% vs. ICP monitoring group, 32.6%; P > 0.05). The incidence rate of secondary brain herniation in the ICP monitoring group was significantly lower compared with the control group (10.9% vs. 20.5%, P = 0.04). Six-month mortality was 6.5% in the ICP group and 9.1% in the control group (P < 0.05), and neurological outcome was better in the ICP group compared with the control group (P < 0.05).

**Conclusion:**

The dynamic ICP value may be more sensitive and effective in preventing secondary brain herniation in patients with hemorrhagic stroke compared with guidance directed by clinical signs and radiological indicators.

## Background

Intracranial pressure (ICP) monitoring is widely used as standard care for patients with severe traumatic brain injury, but its use in patients with hemorrhagic stroke has not been rigorously assessed. Currently, hemorrhagic stroke has the highest mortality rate of all stroke subtypes [1]. Hematoma growth is a principal cause of early neurological deterioration [2]. Thus, early warning of hematoma enlargement is critical in clinical settings.

Increased ICP following cerebral hemorrhage is associated with secondary hematoma enlargement, brain herniation, and worse neurological outcomes. The evaluation of ICP can be obtained through several methods including direct detection and indirect assessment according to clinical signs and radiological characteristics. Traditionally, deteriorating clinical signs should prompt a repeat computed tomography (CT) scan to assess the need for urgent neurosurgical intervention. However, this is likely to be delayed by late or inaccurate observation of patients in clinical settings. Direct ICP monitoring allows early detection of pressure changes and can guide treatment of elevated ICP.

Few studies have investigated the relationship between ICP elevation and neurological deterioration in hemorrhagic stroke, and whether ICP monitoring is useful to indicate early hematoma enlargement or brain herniation [1]. Thus, in this study, we investigated the potential effects of continuous ICP monitoring in the warning of hematoma enlargement and occurrence of brain herniation following hemorrhagic stroke.

## Methods

### Study design and patients

This study was a parallel-group trial with randomized assignment to the ICP monitoring group or control group. Patients with hemorrhagic stroke were admitted to the Neurosurgical Department of the Shanghai Pudong New Area People’s Hospital from January 2010 to December 2012. The inclusion criteria in this study were as follows: (1) The aetiology for hemorrhages was hypertension; (2) All patients did not have initial intraventricular hemorrhage and brain herniation when admitted; (3) All patients were enrolled within 6 h of post-hemorrhagic stroke. We excluded patients with previous cerebrovascular diseases or taking oral anticoagulants. Three-dimensional CT and magnetic resonance imaging scans were used to rule out aneurysms, arteriovenous malformations, and amyloid hemorrhages.

### Group assignments and procedures

Randomization sequences were computer generated by Excel random number generation. Patients in the ICP group underwent ICP monitoring implantation under general anesthesia (Codman, Johnson & Johnson, Raynham, MA, USA). The ICP sensor was inserted into the anterior horn of the lateral ventricle, allowing for drainage of cerebral spinal fluid (CSF) [3]. The contralateral side was chosen for external ventricular drain placement to avoid hematomas. Ventricular ICP measurement is considered a 'gold-standard’ method for assessing elevations in global ICP [4]. Both groups were given routine medical therapies including hemostasis, reduction of blood pressure, and diuretic or mannitol treatment according to established guidelines [1,5]. Neurological exams including the pupil test, extremity test and reflex test were conducted in both groups every 8 h during the first three days, and a repeat CT scan was scheduled 24 h after stroke, and the neurological assessment was performed every 24 h thereafter only if no clinical deterioration was noted. This protocol is designed to balance the evaluation frequency between the ICP monitoring group and control group.

When the ICP was > 25 mmHg, mannitol or a diuretic was immediately administered and CSF was drained to lower the ICP; otherwise, the diuretic was given every 8 h/day to maintain the osmotic pressure between 280 and 320 osmol/L. If the ICP was still > 30 mmHg for 15 min or longer, then the patient was required to undergo CT scanning to assess the progression of hematoma. In the control group, the dosage of mannitol was based on neurological signs and physiological measurements.

### Outcomes

The primary outcome, assessed within 1 month after study onset, was a composite of incidence rate of hematoma enlargement and brain herniation. Hematoma expansion was diagnosed if a patient’s repeat CT scan was read as worsening because of new lesions or an increase in the original volume of abnormalities (≥25% increase in the lesion on the first post-injury CT scan) [6]. The volume of hematoma was calculated using the traditional Approximate Bayesian Compressive Sensing (ABCS) method [7]. Brain herniation is shown by a lowered level of consciousness with a Glasgow Coma Scale (GCS) score of 3–8, plus one or both pupils may be dilated and fail to constrict in response to light [8], which is confirmed by a head CT scan. The secondary outcome was neurological status assessed using the Glasgow outcome scale (GOS) at 6 months post-onset. The GOS is a five-point scale with a range of 1 (dead) to 5 (good recovery). The GOS was determined through an outpatient follow-up or a structured telephone interview by staff blinded to patient grouping.

### Protocol approval and patient consent

The study protocol was approved by the local ethics committee of the Shanghai Pudong New Area People’s Hospital. For study registration, written informed consent was obtained before the study began from patient’s relatives. For follow-up by telephone interview, verbal informed consent was also obtained.

### Statistical analysis

All statistical analyses were performed using SPSS version 20.0 (SPSS, Inc., Chicago, IL, USA). P < 0.05 was considered statistically significant. Characteristics of the ICP group and control group were compared using a Chi-square test or unpaired *t*-test in the case of ordinal variables. The incidence rates of hematoma enlargement and brain herniation were compared using the Chi-square test and Fisher’s exact test, and GOS scores were compared using the Wilcoxon rank test.

## Results

### Patient demographics and early injury characteristics

A total of 90 patients with hemorrhagic stroke were enrolled in our study, including 46 patients in the ICP monitoring group and 44 patients in the control group. Detailed demographics of patients are shown in Table 1. Baseline conditions such as age, gender, admitted GCS, time from onset to admission, systolic blood pressure, location of hematoma, and hematoma volume were not significantly different between the two groups (P > 0.05).

**Table 1:** Patient characteristics and baseline parameters

	**ICP monitoring group**	**Control group**	**P**
Patient numbers	46	44	
Age (yrs)	42.5 (20–65)	43 (18–68)	0.12
Gender, male/female	34/12	29/15	0.49
GCS at admission	10 ± 1	11 ± 1	0.92
Time between onset and admission (hours)	2.46 ± 0.58	2.30 ± 0.81	0.58
Systolic pressure (mmHg)	167 ± 16	170 ± 12	0.44
Location of hematoma (Left/Right)	29/17	27/17	0.83
Hematoma volume (mLs)	19 ± 6	21 ± 4	0.67

### Incidence rates of hematoma enlargement and brain herniation post-onset

Hematoma enlargement was found in most patients 3–10 days post-onset. The incidence of hematoma enlargement in the ICP monitoring group (32.6%) was similar to the control group (38.6%) (χ [2] = 4.717, P = 0.76) (Table 2). However, only 10.9% of patients with ICP monitoring developed secondary brain herniation; while around 20.5% of patients without ICP monitoring deteriorated into brain herniation status (P = 0.04) (Table 2). All enrolled patients with brain herniation underwent acute evacuation of hematoma. There were no complications associated with ICP monitoring including hemorrhage or infection in the ICP monitoring group.

**Table 2:** Incidence of hematoma enlargement and secondary brain herniation in ICP and control groups

		**Cases**	
**Group**		**Hematoma enlargement**	**Brain herniation**
Control	44	17	9
ICP monitoring	46	15	5
P		0.76	0.04

### Neurological outcome

Statistical analysis showed that 6-month mortality was 6.5% in the ICP monitoring group and 9.1% in the control group (P = 0.04). Furthermore, neurological outcome was significantly different between the two groups (Z = 7.133, P = 0.03) (Table 3).

**Table 3:** 6-month GOS in ICP and control group

**Group**	**Cases**	**GOS**					**P**
		**5**	**4**	**3**	**2**	**1**	
Control	44	14	9	9	8	4	Z = 7.133
ICP monitoring	46	28	8	4	3	3	P = 0.03

## Discussion

In this prospective study, compared with the control group, the ICP monitoring group experienced a lower incidence of secondary brain herniation within 1-month post-onset. In addition, patients with ICP monitoring had better neurological outcomes compared with those without ICP monitoring. This may indicate the superiority of clinical management guided by ICP monitoring over guidance by neurological examination and serial CT imaging in patients with hemorrhagic stroke.

The expansion of hypertensive intracerebral hemorrhage occurs earlier, and brain edema emerges more gradually. An enlarged hematoma and edema may result in a reduction in intracranial volume reserve followed by a rise in ICP, and further cause dysfunction of cerebral vascular auto-regulation and decreased cerebral perfusion. If not promptly treated, this can lead to severe deterioration. Thus, early amelioration of increased intracranial pressure is critically important for patients with hemorrhagic stroke. However, there are few studies regarding the relationship between the dynamic change in ICP and neurological outcome, and correlation of ICP monitoring with hematoma enlargement and secondary brain herniation.

A sudden increase and fluctuation of blood pressure can easily lead to further bleeding with the formation of an enlarged hematoma, which will also cause increased ICP. In such circumstances, ICP monitoring allows early warning of these pathological changes. During clinical practice, if ICP is > 30 mmHg and lasts at least 15 min, then the patient will undergo immediate CSF drainage, followed by an urgent CT scan to detect hematoma enlargement. After evacuation of the hematoma, the ICP in most patients will decrease to < 20 mmHg, which is an important predictor of clinical recovery.

Although ICP monitoring cannot directly decrease the incidence of brain herniation, patients with ICP monitoring seem to undergo more intensive care from medical staff. In addition, pharmaceutical therapies such as dehydration, anti-hypertension, and hemostatic control can be adjusted more precisely and timely according to the dynamic ICP values compared with those indicated by clinical signs and radiological findings.

Finally, some limitations of our study must be mentioned. First, some studies have suggested that ICP monitoring is associated with an increased chance of developing iatrogenic complications, such as intracranial infection and ventricular hemorrhage [9], but these signs were not observed in our study, especially with regard to intraventricular hemorrhage. Furthermore, this was a single-center report. The validity of our results in a larger population with hemorrhagic stroke needs to be confirmed in future prospective, multicenter studies. Last, ICP monitoring is much more beneficial in patients with intraventricular hemorrhage. Future studies are required to compare the effects of ICP monitoring between patients with intracerebral hemorrhage and intraventricular hemorrhage.

## Conclusion

There are many studies supporting the use of ICP monitoring in patients with traumatic brain injury, but few have reliably evaluated its effect in patients with hemorrhagic stroke. In this randomized controlled study, we found a lower incidence rate of secondary brain herniation in patients with ICP monitoring. Furthermore, these patients had better neurological outcomes compared with those without ICP monitoring. Although our results demonstrate the superiority of treatment based on ICP monitoring over treatment guided by neurological testing and serial CT imaging in improving long-term neurological recovery, the precise selection of candidates for ICP monitoring and long-term follow-up need to be validated in future multicenter, randomized controlled studies.

## Competing interest

The authors declare that they have no competing interests.

## Authors’ contributions

ZJS carried out the collection of clinical data. TWS performed the statistical analyses. ZP drafted the manuscript. FWM conceived of the study, and participated in its design and coordination and helped to review the manuscript. All authors read and approved the final manuscript.

## Authors’ information

Jingsong Zeng and Ping Zheng are co-first author.

## Disclosure statement

All authors have no conflict to report.

## Pre-publication history

The pre-publication history for this paper can be accessed here:

http://www.biomedcentral.com/1471-2253/14/19/prepub
